# A preliminary DTI study showing no brain structural change associated with adolescent cannabis use

**DOI:** 10.1186/1477-7517-3-17

**Published:** 2006-05-09

**Authors:** Lynn E DeLisi, Hilary C Bertisch, Kamila U Szulc, Magda Majcher, Kyle Brown, Arthika Bappal, Babak A Ardekani

**Affiliations:** 1 The Center for Advanced Brain Imaging, The Nathan S. Kline Institute for Psychiatric Research, Orangeburg, New York, USA; 2 The Department of Psychiatry, New York University School of Medicine, New York, New York, USA

## Abstract

Analyses were performed on brain MRI scans from individuals who were frequent cannabis users (N = 10; 9 males, 1 female, mean age 21.1 ± 2.9, range: 18–27) in adolescence and similar age and sex matched young adults who never used cannabis (N = 10; 9 males, 1 female, mean age of 23.0 ± 4.4, range: 17–30). Cerebral atrophy and white matter integrity were determined using diffusion tensor imaging (DTI) to quantify the apparent diffusion coefficient (ADC) and the fractional anisotropy (FA). Whole brain volumes, lateral ventricular volumes, and gray matter volumes of the amygdala-hippocampal complex, superior temporal gyrus, and entire temporal lobes (excluding the amygdala-hippocampal complex) were also measured. While differences existed between groups, no pattern consistent with evidence of cerebral atrophy or loss of white matter integrity was detected. It is concluded that frequent cannabis use is unlikely to be neurotoxic to the normal developing adolescent brain.

## Introduction

Cannabis abuse is considered a major public health problem worldwide [1] and moreover, cannabis is the most frequent drug of abuse among adolescents [2]. Despite being illegal in many countries it is easily obtainable and even homegrown. Most individuals who frequently use it report a mild euphoric feeling and sense of wellbeing, a reason for its continual popularity. However in some individuals, frequent use has adverse consequences and can even lead to psychotic symptoms (reviewed in [3]). The adolescent brain may be particularly sensitive to any effects it may have because of the continued growth and differentiation of higher brain cortical centers throughout these specific years of life [4].

In the early 1970's a controversial report was published in the Lancet concluding that cannabis caused cerebral atrophy as evidence in a pneumoencephalography study of a small group of male users [5]. This was followed by several CT studies in the 1970's and 80's that refuted these findings [6-9]. More recently, two MRI studies [10,11] reported no difference in gray or white matter vollumes, CSF, or hippocampus volumes in heavy cannabis users compared with non-users. However, specific measures of white matter change were not examined and the subjects studied were not necessarily adolescent cannabis users. Since cannabis use changes the density of cannabinoid -1 receptors in the brain [12], it is possible that this density alteration could be associated with volume loss as detectable by MRI in cannabinoid receptor rich brain regions, such as temporal cortex. In one study, Nerve Growth Factor (NGF), which is known to be released after neuronal damage, was found to be increased in serum subsequent to cannabis use and could be evidence of neurotoxicity [13]. While there is no convincing evidence that cannabis is neurotoxic, the establishment of new cortical connections and growth of axons that normally occur during adolescence could be disrupted by frequent cannabis use. Diffusion tensor imaging (DTI) is particularly useful to test this hypothesis.

DTI is an MRI technique that measures the Brownian motion or free diffusion of water [14]. Images obtained using DTI are used to estimate a diffusion tensor, **D**, for each voxel, which models the water diffusion. The diffusion tensor can be further processed to compute the fractional anisotropy (FA), a normalized scalar measure of the degree of diffusion anisotropy within a voxel. FA reflects the degree of fiber organization, fiber directional coherence, or fiber integrity. White matter abnormalities with axonal disorganization might therefore be expected to have decreased FA. DTI may also be used to assess regional volume deficits using ADC-based morphometry (ABM; [15]), although this association has never been directly validated. Voxelwise ADC is computed as the trace of the diffusion tensor **D **and compared between groups. ABM relies on the assumption that reductions in brain volume are accompanied by commensurate increases in the local volume of CSF. Because the ADC of CSF is greater than that of brain parenchyma, one would expect an increase in ADC in voxelwise group analyses in regions with volume deficits.

Thus, the current report uses DTI to address whether cannabis affects normal brain structures and their white matter integrity during adolescence.

## Methods

Young adults (between the ages of 17 to 30) were recruited for this study either by use of a research normal volunteer pool at The Nathan S Kline Institute for Psychiatric Research (NKI) or by direct advertisement. The NKI volunteer pool was established in the research outpatient department to develop a cohort of community individuals who are available for participation in multiple human research studies. They are ascertained through advertisement in the local communities surrounding the institute. Others were recruited directly by advertisements for normal participants for research studies. Individuals were not recruited based on whether they had a history of cannabis use or not and advertisements did not mention cannabis. Cannabis users were consecutively admitted into the study if the cannabis use began prior to the age of 18 and consisted of use more than 21 times in any single year (N = 10; 9 males, 1 female, mean age 21.1 ± 2.9, range 18–27). This cut-off was chosen based on the cut-off already established by the structured interview format used (DIGS, [16]). All 10 positive adolescent users were not current frequent users; but their periods of use ranged from daily for one or more years to 2–3 times per week for 1 or more years during adolescence. Of these, 3 also admitted to having used other illicit drugs as well in the past (not currently) as well as frequent alcohol use in the past, two of them at the time of this study. No others were frequent alcohol users. Control subjects were admitted into the study if they had no illegal substance use, no frequent alcohol use in the past or currently and were not taking any medication for long periods. They were matched one to one to each cannabis user for sex first and then as close as possible to age and to social class (years of education and occupational status) of parents from a group of consecutively obtained controls who had MRI scans performed. The 10 matched non-user controls were 9 males and 1 female, mean age of 23.0 ± 4.4 (range: 17–30). The 10 cannabis users were the first 10 controls obtained who admitted to cannabis use according to the above criteria. No volunteer who had evidence of a psychosis by DIGS interview was admitted into the study. Individuals who had a family history of schizophrenia in a first or second degree relative were eliminated from this analysis because studies of individuals at genetic high risk for schizophrenia within our research program show brain differences compared with controls (unpublished data). All individuals signed written informed consent for participation in this research. The MRI scan protocol was approved by the Center for Advanced Brain Imaging at NKI and the Institutional Review Board. All subjects signed written informed consent for participation in an MRI study.

MRI scans were performed on a 1.5 T Siemens Vision system (Erlangen, Germany). Image sequences acquired included: a 3D magnetization-prepared rapid gradient echo (MPRAGE) image (TR/TE = 11.6/4.9 ms, flip angle 8°, 256 × 256 × 172 matrix size, 1.20 × 1.20 × 1.20 mm^3 ^voxel size), a T2-weighted spin-echo image (TR/TE = 5000/90 ms, 24 slices, 5 mm slice thickness, no gap, 256 × 256 matrix size, 0.88 × 0.88 mm^2 ^pixel size), diffusion weighted images (TR/TE = 6000/100 ms, b-value = 1000 s/mm^2^, 8 non-collinear gradient orientations, 7 averages, 19 slices, 5 mm slice thickness, no gap, 128 × 128 matrix size, 2.5 × 2.5 mm^2 ^pixel size), and one image without diffusion sensitizing gradients (b = 0).

The processing steps for voxelwise analyses of the FA and ADC maps were exactly the same. The 8 DTI volumes with (b = 1000 s/mm^2^) and the one volume without diffusion gradients (b = 0) were used to estimate a second order symmetric diffusion tensor **D **at each voxel [14], from which the FA and ADC values were computed. The computed FA and ADC maps were spatially normalized to a standard space to facilitate voxelwise statistical analysis. Details of the registration process are given elsewhere [17].

To assess the between group differences in the FA or ADC maps of the subjects with and without cannabis use, two-tailed voxel-wise independent samples student's t-tests were used. The obtained t-map was then threshold at P < 0.01 to find voxels where the FA or ADC values significantly differed between the groups. To reduce the false-positive rate, clusters of size 200 mm^3 ^or greater in the thresholded image were retained (see [17]).

Volumetric measurements were obtained of whole brain, temporal lobe, the superior temporal gyrus, hippocampus and amygdala as a complex and cerebral ventricles These structures were chosen because they most often are related to psychotic experiences and memory. Since cannabis can occasionally lead to psychotic experiences and transient cognitive problems, focusing on these brain regions seemed warranted. For all volumetric measurements T1-weighted MPRAGE images were used. The T1 images were resliced into 2 image sets: One set to be used in automated tissue segmentation for white matter FA tissue masking; these co-registered images were resliced using the pixel dimensions of the FSE images (2.5 mm^3 ^× 40 slices). The segmented FA masks were used for global white matter FA determinations; The second image set was used for the evaluation of gray matter volumetric measures and were resliced using the original pixel sizes (1.2 mm^3 ^× 172 slices). The slice editing software 3D Slicer [18] was used for localization of anatomical landmarks, manual segmentation of anatomical regions of interest (ROIs), and the creation of regional ROIs from grey/white segmented images (measurement reliability ranged from an ICC of 0.99 for ventricles and hemispheres, .93 for left and .97 for right temporal lobes, .91 left and .93 for right superior temporal gyrus and .85 for left and .89 right amygdala-hippocampal complex). This program permits optimized visualization of anatomical MRI data and allows for manual segmentation in all image planes as well as landmark identification in multiple planes simultaneously. 3D Slicer is freely available, open-source software available for a variety of operating systems including Linux and Windows. Atlases [19,20], depicting three-dimensional sectional anatomy and describing variations in cerebral sulcal anatomy, were also used as aids. All manual delineations were performed separately for each hemisphere on coronal slices containing the structure of interest. Sagittal and axial views were used for anatomical reference. Whole brain measurements included both the cerebellum and lateral ventricles, and were solely used to control for brain size in testing hypotheses about specific structures. These data were analyzed for group differences using an ANCOVA controlling for whole brain size and age. Ventricular size was considered an indicator of overall atrophy.

Anatomical locations for any statistically significant differences were determined using AFNI [21]. AFNI provides Talairach coordinates for a spatially registered image. These images are then checked against standard Talairach atlases for corresponding anatomical structures [19].

## Results

### Volumetric measurements

Table 1 shows no significant change in any measured brain structures in the cannabis users compared with controls.

**Table 1:** Volumetric measurements in cm-3 +/- standard deviations. An ANCOVA was conducted to control for whole brain volume and age for each structure. The temporal lobe, superior temporal gyrus and hippocampus/amygdale are gray matter segmented volumes only.

Structure Volume (in cm-3)	Cannabis Users (N = 10)	Cannabis Non-Users (N = 10)	F	P<
Whole Brain	1529.41 +/- 109.59	1452.25 +/- 113.35	-1.55	0.14

Lateral Ventricles				
Left	7.28 +/- 3.46	5.71 +/- 2.24	1.13	0.35
Right	6.47 +/- 4.00	5.05 +/- 2.05	1.02	0.38

Amygdala-Hippocampus				
Left	6.10 +/- .75	5.70 +/- .81	1.03	0.38
Right	6.23 +/- 1.00	5.97 +/- .90	0.83	0.46

Temporal Lobe				
Left	70.86 +/- 5.41	69.31 +/- 9.84	1.47	0.24
Right	69.69 +/- 5.04	70.85 +/- 6.60	2.281	0.15

Superior Temporal Gyrus				
Left	13.38 +/- 1.22	13.44 +/- 1.94	0.01	0.99
Right	12.16 +/- 1.64	12.28 +/- 1.28	0.09	0.92

### Voxelwise analyses

**Figure 1:**
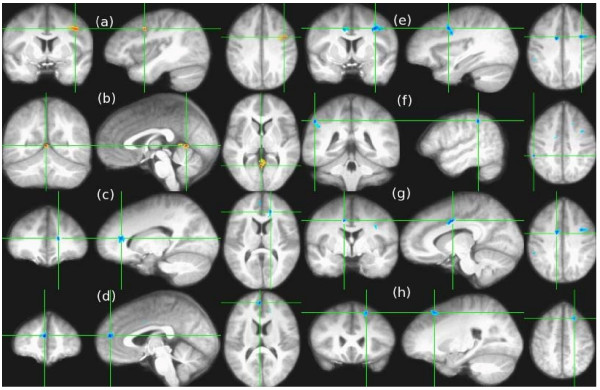
This figure illustrates significant differences in cannabis users relative to non-users in both ADC and FA. Coronal, sagittal and axial views (moving from left to right, respectively) illustrate that the apparent diffusion coefficient (ADC) for adolescent cannabis users (N = 10) was significantly (p < 0.01, cluster size >200 mm^3^) lower relative to non-cannabis users (N = 10) in left middle frontal gyrus **(a)**: Talairach coordinates -41 [L],3 [A],36 [S], and posterior to the right posterior cingulate **(b)**: 2 [R],-54 [P],4 [S] (shown in orange superimposed on the average registered MPRAGE images from all 20 subjects), and that the fractional anisotropy (FA) was significantly higher (shown in blue) in cannabis users in the left anterior cingulate **(c)**: -16 [L],32 [A],11 [S], right medial frontal gyrus **(d)**: 3 [R],46 [A],18 [S], left precentral gyrus **(e)**: -32 [L],1 [A],33 [S], right inferior parietal **(f)**: 50 [R],-38 [P],39 [S], right cingulate gyrus **(g)**: 10 [R],-3 [P],35 [S], and left superior frontal gyrus **(h)**: -20 [L],21 [A],45 [S].

There were two regions (p < 0.01, cluster size > 200 mm^3^) where the ADC was reduced in cannabis users relative to non-users. These are shown in Fig. 1, parts (a) and (b). There were no regions where the ADC was significantly increased. There were six regions (p < 0.01, cluster size > 200 mm^3^) where the FA was increased in cannabis users relative to non-users. These regions are shown in Fig. 1, parts (c)-(h). The FA was not reduced in any regions in cannabis users compared with non-users. Because these results did not show any increases in ADC or reductions in FA in cannabis users, we relaxed the significance level to p < 0.05 without a change in the direction of the results. Thus only the p < 0.01 maps are shown.

## Discussion

Adolescence is a time of particular vulnerability for brain maturation. During this period many individuals experiment with illicit substance use and sometimes quite frequently. Some adolescents who abuse cannabis subsequently develop chronic serious psychiatric symptoms, such as schizophrenia (e.g. [22]) and also cognitive deficits [23-25]. However, it has never been shown consistently that cannabis has direct effects on brain development and there are no known reports using more advanced imaging technology such as DTI to examine white matter integrity. Thus the current study was an initial evaluation to determine whether any indication of cortical atrophy or white matter abnormalities could be detected applying these current MRI methods.

Although differences were observed between subjects who used cannabis during adolescence and those who did not, no finding indicated pathological change. Regions of higher ADC, putative evidence of atrophy, were not present, although regions of significantly lower ADC were. While low FA would be indicative of less white matter integrity, particularly with respect to fiber direction, all FA differences in this study were higher values in cannabis users than non-users.

However, one limitation of the current study is its cross-sectional evaluation of subjects reporting on their own former adolescent cannabis use, rather than a longitudinal design following adolescents into adulthood to observe how the brain changes over time or alternatively a cross-sectional study of current cannabis-using adolescents. Pathological effects from prior frequent use may be less detectable in adulthood after time has passed and other changes have taken place to compensate for possible earlier effects of cannabis.

In addition, although we suggest here that the ADC indicates the amount of CSF in extracellular tissue and ventricular space, we have not yet validated this assumption by direct comparisons and thus this view, while logical, remains speculative at present.

Thus, these data lead to the likely conclusion that cannabis use, in at least moderate amounts, during adolescence does not appear to be neurotoxic, although we cannot exclude any adverse effects of heavier amounts than that used by the current subjects. These data are preliminary and need replication with larger numbers of subjects, although they do have implications for refuting the hypothesis that cannabis alone can cause a psychiatric disturbance such as schizophrenia by directly producing brain pathology.
